# Assessing Statistical Anxiety Among Online and Traditional Students

**DOI:** 10.3389/fpsyg.2019.01440

**Published:** 2019-07-04

**Authors:** Marta Frey-Clark, Prathiba Natesan, Monique O’Bryant

**Affiliations:** ^1^Educational Psychology, University of North Texas, Denton, TX, United States; ^2^Atlanta Public Schools, Atlanta, GA, United States

**Keywords:** statistical anxiety, online education, measurement invariance, statistics education, validity

## Abstract

The purpose of this study was to determine whether scores on the Statistical Anxiety Scale (SAS) manifest in the same way for students in online and traditional statistics courses. Tests of measurement invariance indicated that invariance of the two-factor model of the SAS held at every level. Therefore, we compared the statistical anxiety of online and traditional students. Results indicated that online and traditional statistics students reported comparable levels of anxiety with slightly less anxiety in terms of seeking help for traditional students. We concluded that online instruction is a viable form of statistics education at least for undergraduate students enrolled in the social sciences.

Participation in online education has grown rapidly over the past 15 years and is expected to continue growing (Allen and Seaman, 2010). In fact, the New York Times declared the year 2012 as the “year of the MOOC” (massive open online courses, Pappano, 2012). In Fall 2015, 29.8% of the students were enrolled online in postsecondary institutions (NCES, 2015). The online learning consortium report further shows how in addition to education, professional development, and other related sources of knowledge have moved digitally (OLC Report, 2018). Indeed, online courses seem to offer distinct advantages, with being a more convenient and cost-effective alternative to traditional, face-to-face instruction. Researchers have worked to keep pace with the growth in online learning, comparing learning outcomes for students enrolled in online courses with those of students enrolled in traditional courses.

Although several meta-analyses have shown that there was no statistically significant difference between instruction employing technology and traditional instruction (Cavanaugh et al., 2004; Zhao et al., 2005; Jahng et al., 2007), other meta-analyses have found a statistically significant difference between online and traditional instruction (Shachar and Neumann, 2003; Allen and Seaman, 2004; Bernard et al., 2004; Sitzmann et al., 2006; Williams, 2006). In fact, students with low GPAs tend to withdraw more from an online course than from a traditional course and online students tend to persist less in their programs to attain a degree (Jaggars et al., 2013). Jaggars (2014) also reported that students reported having to “teach themselves” in an online class. With respect to performance although there was a statistically significant relationship between course format (online vs. traditional) and failure in the course for English and Math courses, this was not the case for Economics and Humanities courses (Griffiths et al., 2014). Thus, it seems that there is a difference in the relationship between student performance and course format by subject matter.

Given the prevalence of anxiety in statistics courses that are perceived to be challenging, several researchers have compared performance outcomes for students enrolled in online and traditional statistics courses. Some authors have reported no difference between the two class formats (McLaren, 2004; Dotterweich and Rochelle, 2012), while one study found a difference favoring traditional instruction (Scherrer, 2011). McLaren (2004) found no statistically significant difference in the grades earned by online and traditional statistics students who completed their course; however, the researcher did find that online students demonstrated a greater tendency to drop the course or “vanish,” failing to take part in assignments and exams despite remaining on the roster. Similarly, Dotterweich and Rochelle (2012) found that students enrolled in online, traditional, and televised instruction statistics courses earned similar grades; however, when the researchers isolated students who were repeating the course, they found statistically significant differences in performance favoring traditional students. By contrast, Scherrer (2011) found that when GPA, class format, and student major were included in a regression equation, class format was a statistically significant predictor of final grades, with traditional students outperforming online students.

Despite a growing body of literature comparing the performance of online and traditional statistics students, there remains a dearth of research comparing the statistical anxiety of online and traditional statistics students. Statistical anxiety is defined as “feelings of anxiety encountered when taking a statistics course or doing statistical analysis; that is, gathering, processing and interpreting data” (Cruise et al., 1985, p. 92). Statistical anxiety is a well-documented reality for statistics students (Onwuegbuzie et al., 2010; Chew and Dillon, 2014), and high statistical anxiety has consistently been associated with lower performance outcomes (Bell, 2001, 2003; Onwuegbuzie, 2004; Galli et al., 2008; Macher et al., 2012). In light of the mixed findings regarding the performance of traditional and online statistics students, as well as the documented relationship between statistics anxiety and statistics performance, it may be useful to examine the relationship between statistics anxiety and class format.

DeVaney (2010) administered a statistical anxiety pretest and posttest to traditional and online graduate students, reporting that online students had higher anxiety at the beginning of the course, but there was no difference in student anxiety at the end of the course. However, DeVaney’s research operated on the assumption that measurement instrument operationalized statistical anxiety in the same way for online and traditional students. Given that previous research has identified situational antecedents to statistical anxiety (Onwuegbuzie and Wilson, 2003), it would seem that the distinct environments of traditional and online students may lead to distinct operationalization of the construct. Thus, a test of measurement invariance is a necessary foundation for future research before comparisons across traditional and online student groups can be conducted.

Measurement invariance tests the equivalence of constructs across groups along four prescribed levels (see Mellenbergh, 1989; Meredith, 1993; Vandenberg and Lance, 2000). A configural invariance model is used to test if the factor structure is defined identically across groups. Once this is established, a metric or factorial invariance model tests the equivalence of factor loadings across groups in addition to identical factor structure. Upon establishing metric invariance, a scalar invariance model is used to test if the factor structure, loadings, and item intercepts are identical across groups. Finally, an error variance invariance model is used to test if the factor structure, loadings, item intercepts, and item error variances are identical across groups. Factor means and variances may be compared only when all these levels of invariance are established. Lack of measurement invariance indicates that group-specific attributes unrelated to the latent constructs contaminate the way a person belonging to a group responds to an item (Meredith, 1993; Little, 1997). In other words, a lack of measurement invariance means that given the same factor score, individuals from different groups will have respond differently to a given item. Thus comparisons of factor scores, means, and variances in such a situation are invalid.

## Measuring Statistical Anxiety

In a review of literature on statistical anxiety, Chew and Dillon (2014) identified six extant scales, but the authors only recommended use of the Statistics Anxiety Rating Scale, or STARS (Cruise et al., 1985), and its abbreviated alternative, the Statistical Anxiety Scale, or SAS (Vigil-Colet et al., 2008). The STARS is the most widely used and well-known scale (Chew and Dillon, 2014). However, Vigil-Colet et al. (2008) criticized the STARS for its length and some of its content, which prompted their development of the SAS. The SAS has 24 items and is comprised of three subscales derived from the STARS anxiety subscales: Examination Anxiety (eight items), Interpretation Anxiety (eight items), and Asking for Help Anxiety (eight items). Examination Anxiety refers to anxiety experienced while taking a statistics test. Interpretation Anxiety refers to anxiety experienced while attempting to derive meaning from statistical formulas and output. Asking for Help Anxiety refers to anxiety experienced while requesting help of a peer, a tutor, or a professor. Each item of the SAS details a specific task, prompting respondents to indicate the level of anxiety associated with the task on a 5-point Likert-type scale ranging between *no anxiety* and *very much anxiety.*

Vigil-Colet et al. (2008) administered a Spanish version of the SAS to a sample of undergraduate students (*n* = 159) enrolled in statistics courses in Spain. An Exploratory Factor Analysis (EFA) verified the intended three-factor structure, with each item loading on its intended subscale. Shortly after the development and validation of the Spanish version of the SAS, Chiesi et al. (2011) administered an Italian version of the SAS to a sample of students (*n* = 512). A confirmatory factor analysis (CFA) confirmed the previously validated three-factor model, with the addition of correlated errors between two similarly phrased items on the Asking for Help subscale. Chiesi et al. (2011) also conducted measurement invariance tests across samples of Italian and Spanish students and reported that strict invariance of the modified three-factor model was tenable across both samples.

Following the validation of the three-factor Spanish SAS (Vigil-Colet et al., 2008) as well as the Italian SAS (Chiesi et al., 2011), O’Bryant (2017) investigated the factor structure of the English version of the SAS. After pilot-testing, she modified the items thus: Many revisions involved changing one word such as replacing *doing* to *completing* in items such as *doing a final exam in a statistics course* to *completing a final exam in a statistics course.* Other examples of changes included changing the word tutor to teacher to reflect the teaching system and terminology in the United States. O’Bryant administered the English version of the SAS to a sample of undergraduate students (*n* = 323) majoring in the humanities and enrolled in statistics courses throughout the United States. A CFA of the previously validated three-factor model indicated poor model fit ($\chi_{SB}^{2}$ = 153.46, df = 71.12, *p* < 0.001, RMSEA = 0.106, CFI = 0.838, SRMR = 0.073). Examination of residual correlations revealed that the residuals of the seven items on the Interpretation subscale were highly correlated with those of the items within the subscale, as well as with items on the other two subscales. Thus, O’Bryant (2017) eliminated the Interpretation subscale from the model. Eliminating the interpretation factor was not only warranted according to factor analytic output, but also seemed conceptually justifiable, given that taking an exam and asking for help are discrete tasks while interpreting numbers is not.

Further examination of residual correlations revealed that one item on the Examination Anxiety subscale and one item on the Asking for Help subscale could be eliminated due to redundancy with other items. Finally, the residuals for four items (items 1, 4, 13, and 20) on the Examination Anxiety scale were allowed to correlate, given the similarity in their wording. The resulting model had two factors, Examination Anxiety and Asking for Help Anxiety, with seven items loading on each factor and correlated errors for four items on the Examination Anxiety factor. This modified two-factor model fit the data well ($\chi_{SB}^{2}$ = 49.37, df = 38.13, *p* = 0.105, RMSEA = 0.076, CFI = 0.959, SRMR = 0.035) and was retained. We extend O’Bryant (2017) validation study to validating the factors across the online and traditional samples using measurement invariance.

The purpose of the present study is to determine whether scores on O’Bryant (2017) modified two-factor model of statistical anxiety are operationalized in the same way for traditional and online statistics students. If measurement invariance is established, an additional purpose of the present study is to compare the latent scores on the Exam Anxiety subscale and the Asking for Help Anxiety subscale for online and traditional students.

## Materials and Methods

Institutional Review Board of the University of North Texas approved the study. A two-stage sampling procedure was used. First, simple random sampling without replacement was used to randomly select institutions with social science programs to participate in the study. Second, network sampling was used to ask instructors of statistics for social science courses to pass along the research opportunity to their students. The goal was to recruit participants similar to those used in previous validation studies (Vigil-Colet et al., 2008; Chiesi et al., 2011) for comparison purposes. Data were collected online using qualtrics. Informed consent was obtained from participants who were all 18 years of age or above by asking them to click on a page that explained the study, the duration of the survey, and letting them know of the anonymity that would be maintained with the data. If they agreed to participate they could continue answering the questions by clicking on an appropriate button, else they could exit the survey. Participants were undergraduate students (*n* = 323) who were majoring in the social sciences and were enrolled in a statistics course. However, data screening revealed that 21 respondents took an online-traditional hybrid course, and seven respondents did not indicate their class format. Because we were only interested in online and traditional groups students, and the hybrid group was too small for analysis, these cases were dropped from the dataset, leaving 295 cases with online (*n* = 52) and traditional (*n* = 243) students. Respondents in the final dataset were predominantly female (75%), predominantly white (59%), and predominantly freshman (38%), with ages ranging from 18 to 63 years (*M* = 20.64, SD = 5.37).

## Results

### Screening

The data were screened for outliers, assumptions of normality, and missing values prior to analysis. There were no outliers identified. Examination of frequency data on each item revealed severely peaked distributions, indicating that scores on the 5-point Likert-type scale were ordinal; thus, all subsequent analyses utilized non-parametric tests. Frequency data for missing values revealed a somewhat consistent distribution of missing data, with 0.3–4.7% missing per variable. Given the small percentage missing per variable and the spread of missingness across variables, data were assumed to be missing completely at random (MCAR) and were estimated *via* Mplus’ default estimation for ordinal outcomes with covariates, making use of all available data to estimate missing values.

### Reliability

Internal consistency of the modified two-factor SAS was measured with Cronbach’s *α* for each class format. The *α* coefficients for the online class format were as follows: Total = 0.903, Exam Anxiety Subscale = 0.903, and Asking for Help Anxiety Subscale = 0.880. The *α* coefficients for the traditional class format were as follows: Total = 0.914, Exam Anxiety Subscale = 0.886, and Asking for Help Anxiety Subscale = 0.922. The entirety of the modified two-factor SAS and its subscales were deemed to have high internal consistent for each class format (Nunnally, 1978; Nunnally and Bernstein, 1994). McDonald’s (1999) omega was computed to be 0.94 for the online class format and 0.84 for traditional class format.

### Invariance Testing

We used Mplus version 7.6 with means and variance adjusted weighted least squares (WLSMV) estimation to test the measurement invariance of the SAS for online and traditional statistics students. WLSMV is a robust weighted least squares estimator that has been recommended for ordinal level data with a sample size greater than 200 (Muthén et al., 1997, unpublished; Rhemtulla et al., 2012). Because the data were ordinal, WLSMV calculates threshold parameters for each response variable to estimate the latent, continuous response indicators that correspond with each item of the SAS. Response indicators were scaled *via* theta parameterization, fixing the variance of each latent indicator to 1 in the reference group.

When comparing nested models, we used *χ*^2^ difference tests to evaluate between-model statistical significance, with a statistically significant result indicating non-invariance across models. However, given the sensitivity of *χ*^2^ to sample size, an *a priori* decision was made to supplement the *χ*^2^ model testing parameters with differences in the Comparative Fit Index (CFI) and the Root Mean Square Error of Approximation (RMSEA), per Chen’s (2007) criteria. Thus, the criteria for rejecting model invariance included the joint decision rules of (1) a statistically significant *χ*^2^ difference (*p* < 0.05); (2) a change in RMSEA ≥ −0.005; and (3) a change in CFI ≤ 0.010. Note that Chen’s (2007) criteria for a change in Standardized Root Mean Square Residual (SRMR) were not included because Mplus does not calculate SRMR when using WLSMV estimation to evaluate a model with covariates.

Analysis began with a confirmatory factor analysis (CFA) for each group, confirming that the O’Bryant (2017) modified two-factor model adequately fit the online group and the traditional group individually. Therefore, measurement invariance was testing by first fitting Model A that is the configural invariance model by fixing the factor structure to be identical across groups. Goodness of fit indices and approximate fit indices were tenable, indicating that the factor structure was the same for each group.

Model B, that is, the metric invariance model, was fitted by retaining the factor structure of Model A and adding constraints on all factor loadings to be equal across groups. Model fit was tenable and was not statistically significantly different from Model A, indicating that the Exam Anxiety factor and Asking for Help Anxiety factor were manifested in the same way across groups. That is, the relationships between these factors and the items that indicate them were identical across online and traditional statistics class formats. Note that the *χ*^2^ values produced by WLSMV estimation are corrected for ordinal level data. As such, the *χ*^2^ difference tests for nested models were also corrected by way of the DIFFTEST option in Mplus.

Model C that is, the scalar invariance was fitted by retaining constraints on factor loadings and adding constraints on item thresholds. For interval level data, testing scalar invariance would involve constraining item intercepts. However, recall that scores on items from the SAS were deemed ordinal; as such, thresholds for response options determine scores on a latent response variable, which indicates the latent factor. Thus, scalar invariance requires each threshold for each indicator to be equal across groups. Fit indices for Model C were tenable, and the fit was not appreciably worse than Model B. Therefore, the scalar invariance model was retained.

Finally, Model D, was used to test strict or error variance invariance by fixing all error variances to 1. This test deviated again from invariance testing with interval level data, in which strict invariance is established by constraining the error variances. Recall that the latent response indicators were scaled *via* theta parameterization, fixing each variance to 1 in the reference group. Thus, strict invariance was tested by fixing the latent indicator variances to 1 in both groups. Again, model fit was tenable. The scaled *χ*^2^ difference test reported a statistically significant difference in fit compared with Model C. However, Chen’s (2007) criteria for assessing differences in model fit using CFI and RMSEA did not indicate appreciably worse fit. Model D was retained, and we concluded that the SAS measures the statistical anxiety of students in online and traditional statistics classes identically. See Table 1 for overall and comparative fit indices.

**Table 1:** Values of selected fit statistics for measurement invariance hypotheses for modified two-factor model of statistics anxiety analyzed across online and traditional student samples.

Model	Model name	$\chi_{SB}^{2}$	df	Model comparison	RMSEA	CFI
Online		89.144	71		0.07 [0.0, 0.012]	0.983
Traditional		99.212			0.04 [0.018, 0.058]	0.995
Model A		185.71	142		0.046 [0.024, 0.053]	0.993
Model B	Metric invariance	198.718	154	B vs. A	0.044 [0.023, 0.061]	0.993
Model C	Scalar invariance	239.053	194	C vs. B	0.04 [0.019, 0.056]	0.993
Model D	Error variance invariance	261.041	208	D vs. C	0.042 [0.023, 0.057]	0.992

*CI, confidence interval. All results were computed in Mplus for theta parameterization*.

**Figure 1:**
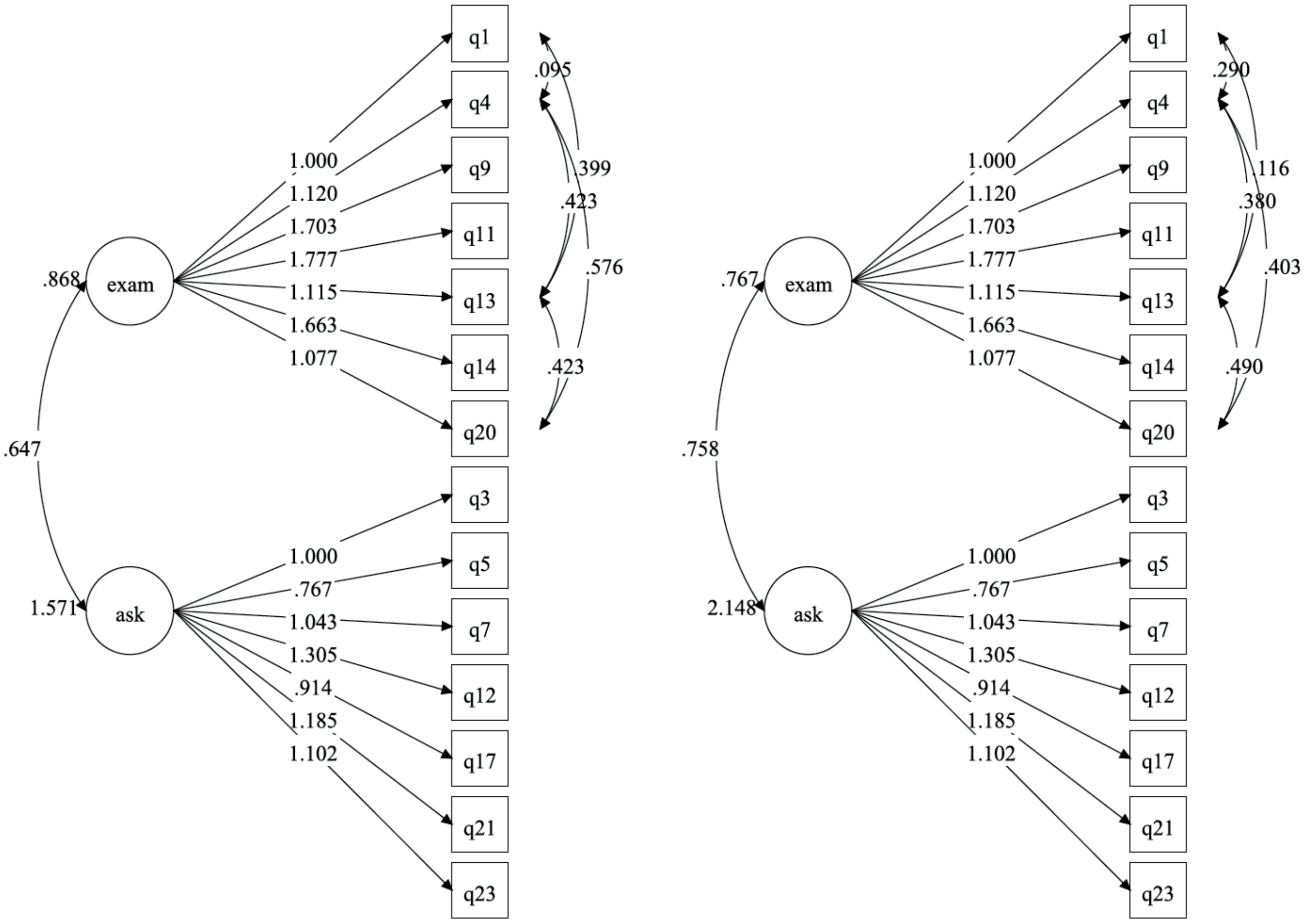
Unstandardized estimates for traditional and online groups.

The unstandardized estimates of Model D for both groups are displayed in Figure 1. We note that we report unstandardized estimates because these are comparable across groups of different sample sizes. Standardized factor loadings for the online group ranged from 0.682 to 0.856; all were statistically significant at the 0.001 level. The correlation between the exam factor and help factor for the online group was 0.554, indicating the factors were related but distinct. Standardized factor loadings for the traditional group ranged from 0.659 to 0.886; again, all loadings were statistically significant at the 0.001 level. The correlation between the exam factor and help factor was 0.591, again indicating the factors were related but distinct.

### Differences in Statistical Anxieties

Having established the measurement invariance of the modified two-factor SAS for online and traditional students, analysis proceeded with the primary purpose of this study: determining by how much the two groups differed in their average scores on the Exam Anxiety subscale and the Asking for Help Anxiety subscale. See Table 2 for the variances and means of each factor for each group. Note that the online group served as the reference group and its factor means were fixed to 0. As such, the factor means listed for the traditional group represent mean differences across groups. The mean difference in Exam Anxiety was 0.048, with online students indicating lower Exam Anxiety. The mean difference in Asking for Help Anxiety was 0.184, with online students indicating higher Asking for Help Anxiety. Cohen’s *d* effect sizes were calculated for both mean differences, revealing effect sizes for Exam Anxiety (*d* = 0.054) and Asking for Help Anxiety (*d* = −0.129) that would be considered a very small effect (Cohen, 1988). Thus, we concluded that online statistics students expressed comparable levels of statistical exam anxiety, but slightly higher levels of asking for help anxiety than traditional statistics students.

**Table 2:** Robust weighted least squares estimates of unconstrained parameters for Model D of statistics anxiety analyzed across online and traditional student samples.

	Online			Traditional		
Parameter	Unstd	SE	Std	Unstd	SE	Std
Exam factor						
Variance	0.87	0.25	1	0.77	0.17	1
Mean	0	–	0	0.05	0.15	0.05
Question factor						
Variance	1.57	0.41	1	2.15	0.35	1
Mean	0	–	0	−0.18	0.21	−0.13
Factor covariance	0.65	0.17	0.55	0.76	0.14	0.59

*Std, Standardized; Unstd, Unstandardized*.

## Discussion

The purpose of the present study was to determine whether the operationalization of statistical anxiety *via* the modified two-factor Statistical Anxiety Scale is the same for samples of online students and traditional students. Previous research has indicated that online statistics students may represent a distinct demographic, being older, with more credit hours earned and more courses repeated than their traditional counterparts (Dotterweich and Rochelle, 2012). Previous research has also indicated online students may possess different intellectual strengths, having higher logical-mathematical intelligence than their traditional counterparts (Lopez and Patron, 2012). If the two populations differ with respect to demographic characteristics and intellectual strengths, it may seem probable that they could differ with respect to the manner in which they report statistical anxiety. However, this was not the case.

Invariance held at every level, indicating that the modified two-factor SAS measures statistical anxiety manifests in the same way for online and traditional statistics students. These findings are further strengthened by the fact that the sample for the present study was drawn *via* random cluster sampling of colleges and universities throughout the United States. Thus, the SAS would appear to be a versatile measure of statistical anxiety. This finding answers Chew and Dillon’s (2014) call to confirm the factor structure of the SAS with diverse samples and provides a foundation for future research using the SAS with classes of varied formats.

Given that the modified two-factor model of the SAS is comprised of only 14 items, and scores on these items are valid for both online and traditional students, statistics instructors may consider administering this instrument to students in order to gauge anxiety and adjust instruction accordingly. Researchers have identified a number of effective interventions, including the use of humor (Pan and Tang, 2004), problem-solving games (D’Andrea and Waters, 2002), and instructor immediacy (Williams, 2006). Thus, the SAS could serve as a diagnostic tool, presenting instructors with student feedback to inform instruction.

An added purpose of this study was to compare mean scores for Exam Anxiety and Asking for Help Anxiety across class formats. Effect size estimates revealed that mean differences were negligible for exam anxiety and a lower asking for help anxiety for traditional students. This is contrary to popular belief that students have lesser inhibitions in reaching out for help when they are learning within the relative privacy and social safety of online education. However, the effect size is too small to make conclusions regarding these differences.

Our findings lend additional support to DeVaney’s (2010) finding that online and traditional students had comparable levels of anxiety upon completion of an introductory statistics course. Furthermore, DeVaney reported that online students had higher statistical anxiety than traditional students at the beginning of the course. Thus, if online students do not appear to carry greater statistical anxiety, as our study suggests, and if the online class format may even soothe statistical anxiety, as DeVaney’s work suggests, then online statistics education seems to present a viable alternative to traditional, face-to-face instruction.

Institutions of higher learning have reported offering online courses in the interest of meeting student demand for flexible scheduling, providing college access to students who may not otherwise have access, making courses more available, and seeking to increase student enrollment (Parsad and Lewis, 2008). As a convenient class format for students, and a cost-effective class format for institutions of higher learning, capitalizing on the pragmatic advantages of online education may allow a greater number of students to access statistics education, and a greater number of institutions to offer statistics education.

A major limitation of the present study is its small sample size. It is recommended that this study be repeated for larger samples so as to address the generalizability of the study. Perhaps administering a pre- and post-survey to examine statistics anxiety before and after taking traditional and online courses is another avenue for future research. Future research might seek to clarify the relationship between class format, statistical anxiety, and performance outcomes. Given the established relationship between statistical anxiety and performance outcomes (e.g., Galli et al., 2008), and the conflicting findings regarding the relationship of class format to performance outcomes (e.g., Scherrer, 2011; Dotterweich and Rochelle, 2012), there exists the possibility that class format and statistical anxiety interact to influence performance outcomes. Examination of all three variables in context may serve to clarify their relationships and inform future instruction. Regardless, insofar as the present study stands, online and traditional statistics students experience similar levels of anxiety, indicating that online instruction is a viable means of delivering statistics education.

## Data Availability

The datasets for this manuscript are not publicly available because the dataset is part of the MOB’s thesis. Covariance matrix may be provided upon request. But the data are subject to confidentiality agreement according to informed consent. Requests to access the datasets should be directed to monique_obryant@yahoo.com.

## Ethics Statement

The institutional review board of the university of North Texas approved this study. Informed consent was obtained from participants before they answered the survey. Vulnerable populations were not involved.

## Author Contributions

MF-C conducted the data analysis and literature review. PN oversaw the project and added conclusion and introduction. MOB collected the data, came up with the instrument, and helped with literature review.

### Conflict of Interest Statement

The authors declare that the research was conducted in the absence of any commercial or financial relationships that could be construed as a potential conflict of interest.

## References

[ref1] Allen I. E. Seaman J. (2010). Class differences: Online education in the United States. (Newburyport, MA: Sloan Consortium).

[ref500] Allen I. E. Seaman J. (2004). Entering the mainstream: the quality and extent of online education in the United States, 2003 and 2004. Newburyport, MA: Sloan Consortium.

[ref2] Bell J. A. (2001). Length of course and levels of statistics anxiety. Education 121, 713–716.

[ref3] Bell J. A. (2003). Statistics anxiety: the nontraditional student. Education 124, 157–162.

[ref501] Bernard R. Brauer A. Abrami P. Surkes M. (2004). The development of a questionnaire for predicting online learning achievement. Distance Educ. 25, 31–47. 10.1080/0158791042000212440

[ref4] Cavanaugh C. Gillan K. J. Kromrey J. Hess M. Blomeyer R. (2004). The effects of distance education on K-12 student outcomes: A meta-analysis. Naperville, IL: Learning Point Associates/North Central Regional Educational Laboratory.

[ref5] Chen F. F. (2007). Sensitivity of goodness of fit indexes to lack of measurement invariance. Struct. Equ. Model. 14, 464–504. 10.1080/10705510701301834

[ref6] Chew P. K. H. Dillon D. B. (2014). Statistics anxiety update: refining the construct and recommendations for a new research agenda. Perspect. Psychol. Sci. 9, 196–208. 10.1177/1745691613518077, PMID: 26173253

[ref7] Chiesi F. Primi C. Carmona J. (2011). Measuring statistics anxiety: cross-country validity of the statistical anxiety scale (SAS). J. Psychoeduc. Assess. 29, 559–569. 10.1177/0734282911404985

[ref8] Cohen J. (1988). Statistical power analysis for behavioral sciences. 2nd edn. (Hillsdale, NJ: Lawrence Earlbaum Associates).

[ref9] Cruise R. J. Cash R. W. Bolton D. L. (1985). Development and validation of an instrument to measure statistical anxiety. *Proceedings of the American Statistical Association, Section on Statistical Education, Las Vegas, NV*.

[ref502] D’Andrea L. Waters C. (2002). Teaching statistics using short stories: reducing anxiety and changing attitudes. In: Sixth International Conference on Teaching Statistics, Cape Town, South Africa.

[ref503] DeVaney T. A. (2010). Anxiety and attitude of graduate students in on-campus vs. online statistics courses. J. Stat. Educ. 18. 10.1080/10691898.2010.11889472

[ref10] Dotterweich D. P. Rochelle C. F. (2012). Online, instructional television, and traditional delivery: student characteristics and success factors in business statistics. Am. J. Bus. Educ. 5, 129–138. 10.19030/ajbe.v5i2.6815

[ref11] Galli S. Ciancaleoni M. Chiesi F. Primi C. (2008). Who failed the introductory statistics examination? A study on a sample of psychology students. Paper presented at the 11th International Congress on Mathematical Education, Monterrey, Mexico.

[ref900] Griffiths R. Chingos M. Mulhern C. Spies R. (2014). Interactive online learning on campus: Testing MOOCs and other hybrid formats in the University System of Maryland. (New York: Ithaka S+R).

[ref12] Jaggars S. S. (2014). Choosing between online and face-to-face courses: community college student voices. Am. J. Dist. Educ. 28, 23–28. 10.1080/08923647.2014.867697

[ref13] Jaggars S. S. Edgecombe N. Stacey G. W. (2013). What we know about online course outcomes. NY: Community College Research Center.

[ref14] Jahng N. Krug D. Zhang Z. (2007). Student achievement in online distance education compared to face-to-face education. Eur. J. Open Dist. Online Learn. 10 http://www.eurodl.org/materials/contrib/2007/Jahng_Krug_Zhang.htm

[ref15] Little T. D. (1997). Mean and covariance structures (MACS) analyses of cross-cultural data: practical and theoretical issues. Multivar. Behav. Res. 32, 53–76. 10.1207/s15327906mbr3201_3, PMID: 26751106

[ref16] Lopez S. Patron H. (2012). Multiple intelligences in online, hybrid, and traditional business statistics courses. J. Edu. Online 9. 10.9743/JEO.2012.2.2

[ref504] Macher D. Paechter M. Papousek I. Ruggeri K. (2012). Statistics anxiety, trait anxiety, learning behavior, and academic performance. Eur. J. Psychol. Educ. 27, 483–498. 10.1007/s10212-011-0090-5

[ref18] McDonald R. P. (1999). Test theory: A unified treatment. (Mahwah, NJ: Erlbaum Associates).

[ref19] McLaren C. H. (2004). A comparison of student persistence and performance in online and classroom business statistics experiences. Decis. Sci. J. Innov. Educ. 2, 1–10. 10.1111/j.0011-7315.2004.00015.x

[ref20] Mellenbergh G. J. (1989). Item bias and item response theory. Int. J. Educ. Res. 13, 127–143. 10.1016/0883-0355(89)90002-5

[ref21] Meredith W. (1993). Measurement invariance, factor analysis, and factorial invariance. Psychometrika 58, 525–543. 10.1007/BF02294825

[ref22] NCES (2015). Available at: https://nces.ed.gov/fastfacts/display.asp?id=80 (Accessed August 15, 2018).

[ref23] Nunnally J. C. (1978). Psychometric theory. 2nd edn. (NY: McGraw-Hill).

[ref24] Nunnally J. C. Bernstein I. H. (1994). Psychometric theory. 3rd edn. (NY: McGraw-Hill).

[ref700] O’Bryant M. J. (2017). How attitudes towards statistics courses and the field of statistics predicts statistics anxiety among undergraduate social science majors: a validation of the Statistical Anxiety Scale. ProQuest LLC. Doctoral dissertation, University of North Texas Available online at https://search.proquest.com/docview/2009455494

[ref25] OLC Report (2018). Available at: https://olc-wordpress-assets.s3.amazonaws.com/uploads/2019/04/OLC-2018-Annual-Report-Online.pdf (Accessed August 15, 2018).

[ref505] Onwuegbuzie A. J. (2004). Academic procrastination and statistics anxiety. Assess. Eval. Higher Educ. 29, 3–19. 10.1080/0260293042000160384

[ref26] Onwuegbuzie A. J. Leech N. L. Murtonen M. Tähtinen J. (2010). Utilizing mixed methods in teaching environments to reduce statistics anxiety. Int. J. Multiple Res. App. 4, 28–39. 10.5172/mra.2010.4.1.028

[ref27] Onwuegbuzie A. J. Wilson V. S. (2003). Statistics anxiety: nature, etiology, antecedents, effects, and treatments—a comprehensive review of the literature. Teach. High. Educ. 8, 195–209. 10.1080/1356251032000052447

[ref506] Pan W. Tang M. (2004). Examining the effectiveness of innovative instructional methods on reducing statistics anxiety for graduate students in the social sciences. J. Instructional Psychol. 31, 149–159.

[ref28] Pappano L. (2012). The year of the MOOC. https://www.nytimes.com/2012/11/04/education/edlife/massive-open-online-courses-are-multiplying-at-a-rapid-pace.html

[ref29] Parsad B. Lewis L. (2008). Distance education at degree-granting postsecondary institutions: 2006–2007. First look (NCES 2009–044). (Washington, DC: U.S. Department of Education, Institute of Education Sciences, National Center for Education Statistics).

[ref30] Rhemtulla M. Brosseau-Liard P. E. Savalei V. (2012). When can categorical variables be treated as continuous? A comparison of robust continuous and categorical SEM estimation methods under suboptimal conditions. Psychol. Methods 17, 354–373. 10.1037/a0029315, PMID: 22799625

[ref32] Scherrer C. R. (2011). Comparison of an introductory level undergraduate statistics course taught with traditional, hybrid, and online delivery methods. INFOMRS Trans. Educ. 11, 106–110. 10.1287/ited.1110.0063

[ref33] Shachar M. Neumann Y. (2003). Differences between traditional and distance education academic performances: a meta-analytic approach. Int. Rev. Res. Open Dist. Learn. 4, 1–20. 10.19173/irrodl.v4i2.153

[ref34] Sitzmann T. Kraiger K. Steward D. Wisher R. (2006). The comparative effectiveness of web-based and classroom instruction: a meta-analysis. Pers. Psychol. 59, 623–664. 10.1111/j.1744-6570.2006.00049.x

[ref35] Vandenberg R. J. Lance C. E. (2000). A review and synthesis of the measurement invariance literature: suggestions, practices, and recommendations for organizational research. Organ. Res. Methods 3, 4–70. 10.1177/109442810031002

[ref36] Vigil-Colet A. Lorenzo-Seva U. Condon L. (2008). Development and validation of the statistical anxiety scale. Psicothema 20, 174–180. 10.1037/t62688-000, PMID: 18206081

[ref37] Williams S. L. (2006). The effectiveness of distance education in allied health science programs: a meta-analysis of outcomes. Am. J. Dist. Educ. 20, 127–141. 10.1207/s15389286ajde2003_2

[ref507] Zhao H. Seibert S. E. Hills G. E. (2005). The mediating role of self-efficacy in the development of entrepreneurial intentions. J. Appl. Psychol. 90, 1265–1272. 10.1037/0021-9010.90.6.1265 16316279

